# Apraclonidine—An eye opener

**DOI:** 10.3389/fopht.2022.902821

**Published:** 2022-08-09

**Authors:** Fabienne C. Fierz, Leah R. Disse, Christopher J. Bockisch, Konrad P. Weber

**Affiliations:** ^1^ Department of Ophthalmology, University Hospital Zurich, University of Zurich, Zurich, Switzerland; ^2^ Department of Neurology, University Hospital Zurich, University of Zurich, Zurich, Switzerland; ^3^ Department of Otorhinolaryngology, Head and Neck Surgery, University Hospital Zurich, University of Zurich, Zurich, Switzerland

**Keywords:** Apraclonidine test, Horner’s syndrome, ptosis, anisocoria, Muller’s muscle, eyelid aperture

## Abstract

Pharmacological testing with apraclonidine eye drops induces a typical reversal of anisocoria in patients with Horner’s syndrome. Moreover, apraclonidine was observed to have an elevating effect on the upper eyelid in Horner’s syndrome as well as in healthy subjects, which is thought to be mediated by alpha-1 adrenergic receptors present in the Muller’s muscle. We aim to quantitatively investigate the effect of apraclonidine on eyelid position in patients with Horner’s syndrome compared to physiological anisocoria based on infrared video recordings from pupillometry. We included 36 patients for analysis who underwent binocular pupillometry before and after apraclonidine 1% testing for the evaluation of anisocoria. Vertical eyelid measurements were taken from infrared videos and averaged from multiple pupillometry cycles. Receiver operating characteristic curves were calculated to determine the optimal cutoff value for change in eyelid aperture pre- and post-apraclonidine. A decrease of inter-eye difference in the aperture of >0.42 mm was discriminative of Horner’s syndrome compared to physiological anisocoria with a sensitivity of 80% and a specificity of 75%. Our data confirm an eyelid- elevating effect of the apraclonidine test, more pronounced in eyes with a sympathetic denervation deficit. Measuring eyelid aperture on pupillometry recordings may improve the diagnostic accuracy of apraclonidine testing in Horner’s syndrome.

## Introduction

Apraclonidine has largely replaced cocaine as a pharmacological test in patients with Horner’s syndrome by inducing a reversal of anisocoria (1–6). Apraclonidine, being an α2-agonist with weak α1-activity, causes a slight constriction of healthy pupils. Its dilating effect on the miotic pupil in Horner’s syndrome results from denervation hypersensitivity to α1-adrenergic stimulation.

Moreover, apraclonidine has been observed to increase eyelid aperture in Horner’s syndrome (5, 7), in botulinum toxin–induced ptosis (8), and in healthy subjects (9) presumably by an α1-receptor–mediated contraction of Muller’s muscle. Muller’s muscle, also named superior tarsal muscle, is a smooth muscle with adrenergic innervation, providing 1–3-mm eyelid elevation (10). The sympathetically innervated inferior tarsal muscle is the analogue in the lower eyelid, and its denervation accounts for a slight raise of the lower eyelid.

In a canine upper eyelid model, Yano et al. demonstrated that a selective α1A-adrenoceptor agonist indeed led to sustained Mueller muscle contraction similar to the effect of phenylephrine (11). A predominance of α1-receptors was also confirmed histopathologically in human ptotic eyelid specimens (12). In the same study, an inverse relationship was found between the eyelid elevation response to phenylephrine and the number of α2-receptors. The α2-receptor-mediated effect inhibits the release of norepinephrine at the synapses in the smooth muscle cells, therefore reducing the α1-mediated muscle contraction.

Considering the effect of apraclonidine on eyelid aperture may be particularly helpful as an adjunct in cases with equivocal pupillary response when pharmacological testing is performed. We aim to quantitatively investigate the effect of apraclonidine on eyelid aperture in patients with Horner’s syndrome compared to physiological anisocoria based on infrared video recordings to determine the diagnostic accuracy of the eye-opening effect.

## Materials and methods

Patients referred to the neuro-ophthalmology unit at the University Hospital Zurich for the evaluation of anisocoria from 2019 to 2021 were recruited consecutively for the study. Inclusion criteria were the presence of anisocoria more pronounced in the dark with or without ptosis and age >18 years. Exclusion criteria were previous ocular or eyelid surgery, ophthalmic treatment affecting pupil size or pupil motility, and conditions affecting pupillary reflexes including third cranial nerve palsy and pharmacological mydriasis.

All subjects underwent binocular pupillometry before and after pharmacological testing with apraclonidine 1% eye drops (one drop in each eye). The pupillometry test paradigm consisted of at least four cycles of 4-s light-on (3 log-lux) and 15-s light-off using a programmable desktop pupillometer [DP-2000, Neuroptics, Irvine, CA, USA (13)]. Pupillometry measures were taken from each eye simultaneously and in synchrony.

The diagnosis of Horner’s syndrome was based solely on the response to apraclonidine testing irrespective of any causative lesion, the presence of ptosis, or heterochromia. The pupil size before and after apraclonidine instillation was calculated as the median size from 3 to 4 s after light-off derived from the pupillometry reading. The apraclonidine test was considered positive when the smaller pupil dilated and the larger pupil constricted, leading to a reversal of anisocoria or a convergence of pupil sizes indicative of Horner’s syndrome. We excluded patients assessed at a latency >160 min after apraclonidine instillation, as the latter resulted in a negative correlation between the time elapsed and the effect on eyelid aperture.

The infrared videos recorded during pupillometry were analyzed using custom software (available upon request) written in MATLAB (Matlab 2019b, The MathWorks Inc., Natick, MA, United States). The pupil size in each eye was determined using our previously published method (13). For measuring the vertical palpebral aperture, a vertical line was preset through the center of the pupil. The intersection of the vertical line with the upper and lower eyelid margins was then determined manually, yielding the vertical eyelid aperture. The measurements were taken in a fixed interval within the first 5 s after light-off, avoiding blinks, and were averaged from three-to- four readings from individual pupillometry cycles. The inter-eye difference of vertical lid opening was calculated by subtracting the eye with the smaller pupil from the eye with the larger pupil. A paired t-test and linear correlation were used to compare the change of eyelid aperture and inter-eye difference pre- and post-apraclonidine using a threshold of p<0.05 for statistical significance. The receiver operating characteristic (ROC) curve using MATLAB was calculated to determine the optimal change of difference in eyelid aperture between the two eyes post-apraclonidine to differentiate Horner’s syndrome from physiological anisocoria.

Written informed consent was obtained from all participants. The study was approved by the Zurich cantonal ethics committee, Switzerland (BASEC-Nr. 2016-02151) in adherence to the Declaration of Helsinki.

## Results

Of 38 patients meeting the inclusion criteria, two were excluded due to dermatochalasis obscuring the eyelid margin, making the measurement impossible. The mean time elapsed between apraclonidine application and pupillometry was 57 min (range, 30–152 min). A total of 18 patients had a positive pupillary apraclonidine test, fulfilling the diagnostic criteria for Horner’s syndrome, and 18 patients with a negative apraclonidine test were considered to represent physiological anisocoria.

**Figure 1:**
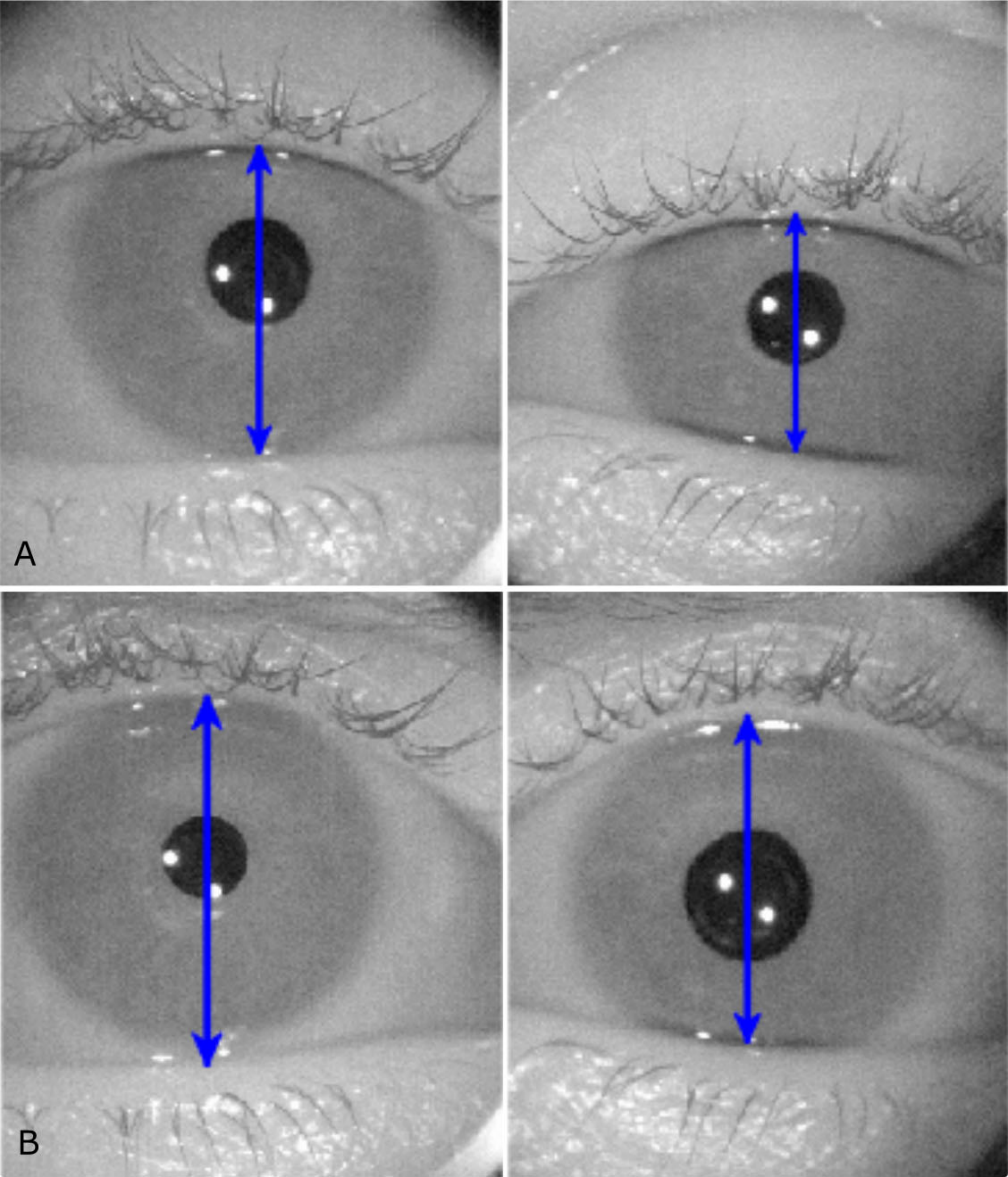
**(A)** Infrared images from pupillometry recordings showing the narrowing of eyelid aperture and miosis on the affected left side in a patient with Horner’s syndrome. **(B)** Approximately 30 min after apraclonidine 1% instillation, the left > right eyelid elevates and anisocoria reverses. Blue arrows correspond to the vertical eyelid aperture.

**Figure 2:**
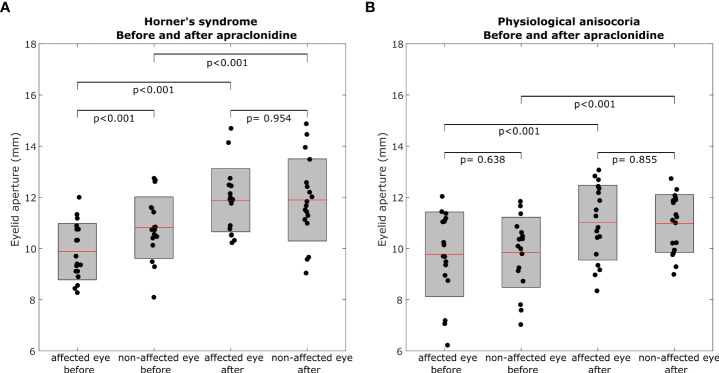
Box plots representing eyelid aperture pre- and post-apraclonidine in Horner’s syndrome **(A)** and physiological anisocoria **(B)**. Central red mark = mean, box boundaries = 25–75th percentiles.

The baseline characteristics of each group are summarized in **Table 1**. **Figure 1** represents an example of left Horner’s syndrome secondary to ipsilateral carotid artery dissection with a positive pupillary apraclonidine test. Eyelid aperture measurements pre- and post-apraclonidine in Horner’s syndrome and physiological anisocoria are summarized in **Table 2** and represented by box plots in **Figure 2**.

**Table 1:** Baseline characteristics.

	Horner’s syndrome		Physiological anisocoria	
Subjects	n = 18		n = 18	
Mean age (years)	52.7 ± 21.9		39.3 ± 11.6	
Female : Male	5:13		10:8	
Pupil diameter 3–4 s after light-off (mean ± SD)	3.3 ± 0.5 mm (affected eye)	4.1 ± 0.5 mm (non-affected eye)	4.1 ± 0.8 mm (side of smaller pupil)	4.5 ± 0.8 mm (side of larger pupil)

**Table 2:** Mean eyelid aperture measured before and after apraclonidine application.

	Horner’s syndrome		Physiological anisocoria	
Side	Affected eye	Non-affected eye	Side of smaller pupil	Side of larger pupil
Eyelid aperture, before apraclonidine (mean ± SD)	9.9 ± 1.1 mm	10.8 ± 1.2 mm	9.8 ± 1.7 mm	9.9 ± 1.4 mm
Eyelid aperture, after apraclonidine (mean ± SD)	11.9 ± 1.2 mm	11.9 ± 1.6 mm	11.0 ± 1.5 mm	11.0 ± 1.1 mm

**Figure 3:**
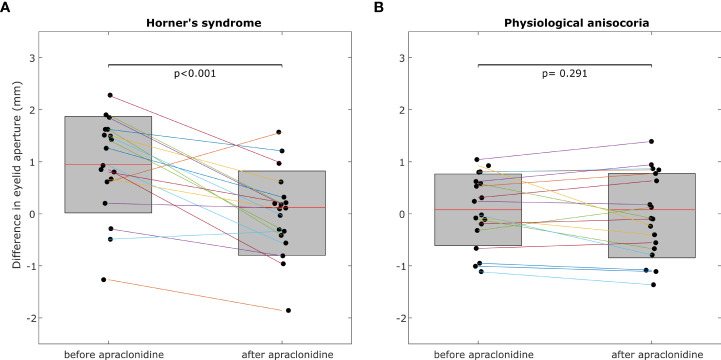
The inter-eye difference of eyelid aperture decreases significantly post-apraclonidine in Horner’s syndrome **(A)** but not in physiological anisocoria **(B)**. Central red mark = mean, box boundaries =25–75th percentiles.

In Horner’s syndrome, the mean eyelid aperture increased by 2.01 ± 1.06 mm (standard deviation) in the affected eye (p<0.001) and by 1.08 ± 0.88 mm in the non-affected eye (p<0.001). The inter-eye difference in eyelid aperture decreased significantly by -0.93 ± 0.80 mm (p = 0.003) (**Figure 3A**), and there was a significant correlation between the initial inter-eye difference and the change induced by apraclonidine (r = 0.567, p = 0.014).

In the group with physiological anisocoria, the mean eyelid aperture increased by 1.23 ± 0.80 mm in the eye with the baseline smaller pupil (p < 0.001) and by 1.12 ± 1.00 mm in the eye with the baseline larger pupil (p < 0.001). The inter-eye difference in eyelid aperture did not change (-0.1 ± 0.44 mm, p = 0.669) (**Figure 3B**), and there was no correlation between the initial inter-eye difference and the change induced by apraclonidine (r=0.013, p=0.958).

**Figure 4:**
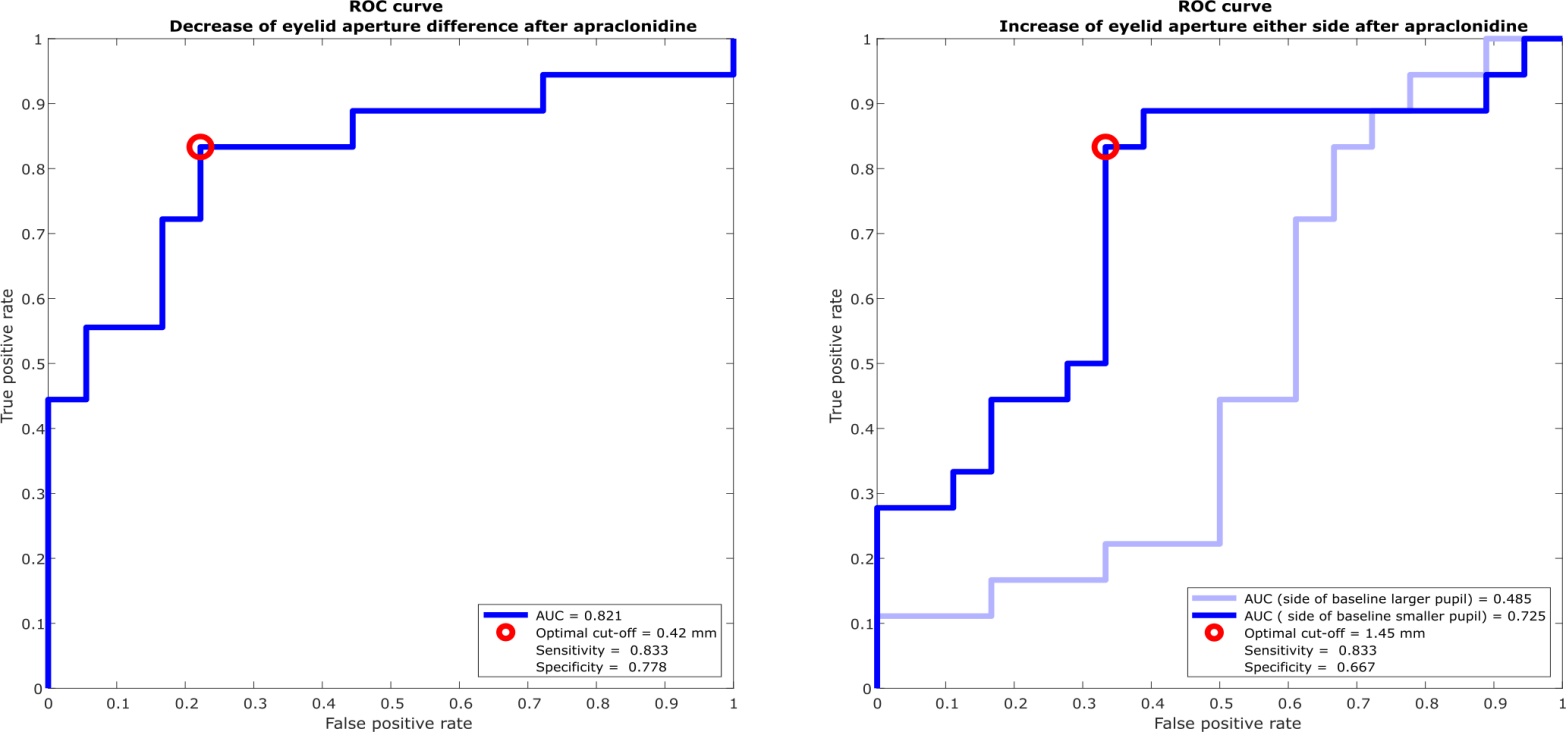
Receiver operating characteristic (ROC) curves for discriminating Horner’s syndrome from physiological anisocoria. **(A)** The optimal cutoff value for change in inter-eye difference of vertical eyelid opening post-apraclonidine is 0.42 mm with a sensitivity of 83% and specificity of 78%. **(B)** The optimal cutoff value for change in eyelid aperture of the eye with the baseline smaller pupil is 1.45 mm with a sensitivity of 83% and specificity of 67%. The change of eyelid aperture in the eye with the baseline larger pupil is not diagnostically helpful with an area under the ROC curve of nearly 0.5.

ROC curves to define the optimal cutoff value for post-apraclonidine eyelid aperture were calculated either based on change in inter-eye aperture difference (**Figure 4A**) or based on the increase of aperture on the affected (baseline smaller pupil) or non-affected side (baseline larger pupil), respectively (**Figure 4B**). For the inter-eye aperture difference, the area under the ROC curve was 0.73, and the best sensitivity of 80% and specificity of 75% was obtained with a cutoff value of >0.42 mm change in inter-eye aperture difference. When measuring only the eyelid aperture of the affected eye, the resulting area under the ROC curve was 0.72, with a sensitivity of 80% and specificity of 70% for a cutoff value of 1.45mm. For the non-affected eye, the area under the ROC curve was 0.52.

## Discussion

Our data show a diagnostically relevant eye-opening effect of topical apraclonidine. The eyelid aperture increased in all subjects irrespective of the presence of Horner’s syndrome and most significantly in eyes affected by the condition. Apraclonidine does not induce a “reversal of ptosis” in analogy to the “reversal of anisocoria” in patients with Horner’s syndrome, but as the inter-eye difference decreases, a positive apraclonidine test results in a nearly symmetrical eyelid position. Similar to the denervation hypersensitivity to the α1-stimulation of the pupil dilator muscle (1), there may be an upregulation of α1-adrenergic receptors in the Muller’s muscle, leading to the overproportionate response to apraclonidine. It is possible that the response is further enhanced by a downregulation of α2-receptors, reducing their inhibiting effect on sympathetic stimulation. In Horner’s syndrome, the well-recognizable mean increase of eyelid aperture was 2.0 mm in our cohort.

In eyes not affected by Horner’s syndrome, we found an increase of eyelid aperture of approximately 0.9–1.0 mm, similar to the effect of 0.7 mm found in healthy subjects by Kirkpatrick et al. (9) Because upper eyelid height was measured relative to the central pupillary light reflex in their study, the results are likely to differ slightly as the contribution of the inferior tarsal muscle to total eyelid aperture is not taken into account. To our knowledge, no studies have addressed the effect of apraclonidine on ptotic eyelids in the context of parasympathetic denervation (third nerve palsy), ocular myasthenia gravis, or levator dehiscence so far. It remains unanswered whether these disorders influence the adrenergic receptor expression in the Muller’s muscle and if, as a consequence, apraclonidine might be useful in the differential diagnosis.

We found that comparing the inter-eye difference pre- and post-apraclonidine and measuring the affected eye alone, i.e., only the eyelid on the side of the smaller pupil, yields similar results (area under the ROC curve 0.821 versus 0.725, respectively). As expected, measuring the aperture of the non-affected eye, that is, the eye with the baseline larger pupil, does not help to distinguish Horner’s syndrome from physiological anisocoria (area under the ROC curve close to 0.5). The inter-eye difference is less prone to influencing factors on eyelid opening, such as general alertness or possible squinting due to photophobia or dry eye sensation, which would affect both eyes equally. A decrease of inter-eye difference by more than 0.42 mm after apraclonidine instillation is suggestive of Horner’s syndrome with a sensitivity of 83% and specificity of 78%. Combining the analysis of eyelid aperture with the change of pupil size after pharmacological testing with apraclonidine is expected to increase the diagnostic accuracy even further. Manual determination of eyelid aperture by an unmasked investigator may constitute a source of confirmation bias in our study, which was minimized by averaging several measurement cycles.

It was previously suggested that apraclonidine may be useful to correct inadvertent, transient botulinum toxin–induced ptosis (8). It remains unknown whether regular apraclonidine application would represent a long-term treatment option for ptosis in Horner’s syndrome. A high rate of tachyphylaxis occurs when apraclonidine is used as an intraocular pressure–lowering agent beyond 3–6 weeks of use (14), and the same might apply to its effect on smooth muscle.

Precise measurement of eyelid aperture in clinical practice is hardly possible without image documentation. Performing the measurements on video recordings offers a feasible approach amenable to the clinical routine if pupillometry is performed. Measuring the effect of apraclonidine on the eyelids may be particularly useful when pupillary measurements are unreliable, such as in post-traumatic or postoperative pupillary damage. When used in addition to analyzing the effect of apraclonidine on the pupillary size, measuring the effect on eyelid aperture may further increase the diagnostic accuracy in evaluating Horner’s syndrome.

## Data availability statement

The raw data supporting the conclusions of this article will be made available by the authors, without undue reservation.

## Ethics statement

This study was reviewed and approved by Kantonale Ethikkommission Zürich, Switzerland. The patients/participants provided their written informed consent to participate in this study.

## Author contributions

FF: study design, data collection, data analysis, and paper writing; LD: data collection, data analysis, and paper revision; CB: data analysis and paper revision; KW: study design, data analysis, and paper revision. All authors contributed to the article and approved the submitted version.

## Funding

The authors received support from the Swiss National Science Foundation (SNSF 320030_166346), the Betty and David Koetser Foundation for Brain Research, Zurich, Switzerland, and the Uniscientia Stiftung, Vaduz, Liechtenstein. The first author was a recipient of the Career Development Grant ‘Filling the Gap’ 2020–2021, Faculty of Medicine, University of Zurich, Switzerland.

## Conflict of interest

The authors declare that the research was conducted in the absence of any commercial or financial relationships that could be construed as a potential conflict of interest.

The reviewer KL declared a shared affiliation with the authors to the handling editor at the time of review.

## Publisher’s note

All claims expressed in this article are solely those of the authors and do not necessarily represent those of their affiliated organizations, or those of the publisher, the editors and the reviewers. Any product that may be evaluated in this article, or claim that may be made by its manufacturer, is not guaranteed or endorsed by the publisher.

## References

[1] Morales J Brown SM Abdul-Rahim AS Crosson CE. Ocular effects of apraclonidine in horner syndrome. Arch Ophthalmol (2000) 118(7):951–4. doi: 10-1001/pubs.Ophthalmol.-ISSN-0003-9950-118-7-ecs90240 10900109

[2] Bremner F. Apraclonidine is better than cocaine for detection of horner syndrome. Front Neurol (2019) 10:55. doi: 10.3389/fneur.2019.00055 30804875 PMC6371044

[3] Brown SM. The utility of 0.5% apraclonidine in the diagnosis of horner syndrome. Arch Ophthalmol (2005) 123(4):578. 10.1001/archopht.123.4.578-a 15824244

[4] Bacal DA Levy SR. The use of apraclonidine in the diagnosis of horner syndrome in pediatric patients. Arch Ophthalmol (2004) 122(2):276–9. doi: 10.1001/archopht.122.2.276 14769608

[5] Freedman KA Brown SM. Topical apraclonidine in the diagnosis of suspected horner syndrome. J Neuroophthalmol (2005) 25(2):83–5. doi: 10.1097/01.wno.0000165108.31731.36 15937427

[6] Chen PL Hsiao CH Chen JT Lu DW Chen WY. Efficacy of apraclonidine 0.5% in the diagnosis of horner syndrome in pediatric patients under low or high illumination. Am J Ophthalmol (2006) 142(3):469–74. doi: 10.1016/j.ajo.2006.04.052 16935593

[7] Garibaldi DC Hindman HB Grant MP Iliff NT Merbs SL. Effect of 0.5% apraclonidine on ptosis in horner syndrome. Ophthalmic Plast Reconstr Surg (2006) 22(1):53–5. doi: 10.1097/01.iop.0000196322.05586.6a 16418668

[8] Wijemanne S Vijayakumar D Jankovic J. Apraclonidine in the treatment of ptosis. J Neurol Sci (2017) 376:129–32. doi: 10.1016/j.jns.2017.03.025 28431598

[9] Kirkpatrick CA Shriver EM Clark TJE Kardon RH. Upper eyelid response to topical 0.5% apraclonidine. Ophthalmic Plast Reconstr Surg (2018) 34(1):13–9. doi: 10.1097/IOP.0000000000000843 27984360

[10] Irene E. Loewenfeld. the pupil: Anatomy, physiology, and clinical applications. 2nd Edition. New York, NY: Butterworth-Heinemann Ltd (1590).

[11] Yano S Hirose M Nakada T Nakayama J Matsuo K Yamada M. Selective alpha 1A-adrenoceptor stimulation induces mueller’s smooth muscle contraction in an isolated canine upper eyelid preparation. Curr Eye Res (2010) 35(5):363–9. 10.3109/02713680903518858 20450248

[12] Skibell BC Harvey JH Oestreicher JH Howarth D Gibbs A Wegrynowski T. Adrenergic receptors in the ptotic human eyelid: Correlation with phenylephrine testing and surgical success in ptosis repair. Ophthalmic Plast Reconstructive Surgery (2007) 23(5):367–71. doi: 10.1097/IOP.0b013e3181462a2e 17881986

[13] Omary R Bockisch CJ Landau K Kardon RH Weber KP. Buzzing sympathetic nerves: A new test to enhance anisocoria in horner’s syndrome. Front Neurol (2019) 10:107. doi: 10.3389/fneur.2019.00107 30846965 PMC6393781

[14] Araujo SV Bond JB Wilson RP Moster MR Schmidt CM Spaeth GL. Long term effect of apraclonidine. Br J Ophthalmol (1995) 79(12):1098–101. doi: 10.1136/bjo.79.12.1098 PMC505348 8562543

